# Green synthesis and characterization of ruthenium oxide nanoparticles using *Gunnera perpensa* for potential anticancer activity against MCF7 cancer cells

**DOI:** 10.1038/s41598-023-50005-7

**Published:** 2023-12-19

**Authors:** Polo-Ma-Abiele H. Mfengwana, Bertrand T. Sone

**Affiliations:** 1 Department of Health Sciences, Central University of Technology, Free State, Park Road, Private Bag X20539, Bloemfontein, 9301 South Africa; 2 Chemistry Department, Faculty of Natural & Agricultural Sciences, University of Pretoria, Pretoria, South Africa

**Keywords:** Biochemistry, Cancer, Drug discovery, Nanoscience and technology

## Abstract

The use of green methods for ruthenium oxide nanoparticles (RuONPs) synthesis is gaining attention due to their eco-friendliness, cost-effectiveness, and availability. However, reports on the green synthesis and characterization of RuONPs are limited compared to other metal nanoparticles. The green synthesis and characterization of RuONPs using water extracts of *Gunnera perpensa* leaves as a reducing agent is reported in this study. The RuONPs were characterized using X-ray diffraction (XRD), Fourier Transform Infrared Spectroscopy (FTIR), Scanning Electron Microscopy (SEM), Transmission Electron Microscopy (TEM), and Ultraviolet spectroscopy (UV–VIS). MTT assay was used to assess the cytotoxicity of the RuONPs against MCF7 and Vero cell lines. X-ray diffraction analysis results revealed the presence of crystalline and amorphous forms of RuONPs, while IR spectroscopy revealed the presence of functional groups associated with *G. perpensa* leaves. SEM showed that the RuONPs consisted predominantly of hexagonal and cuboid-like structures with a considerable degree of agglomeration being observed. The cell culture results indicated a low anticancer efficacy of RuONPs against MCF7 and Vero cell lines, suggesting that RuONPs may not be a good lead for anti-cancer drugs. This study highlights the potential of using green synthesis methods to produce RuONPs and their characterization, as well as their cytotoxicity against cancer cells.

## Introduction

Plant-based medicine has been widely utilized since the ancient days. The World Health Organization (WHO) reports an estimated 80% of people depend on herbal and traditional medicines for health care regulation. Medicinal plants constitute secondary metabolites of medical importance to humans. These secondary metabolites contribute to the pharmacological activity of plants as anticancer agents^1^. In addition, they act as reducing agents during the green synthesis of metal nanoparticles. Nanoparticles play a significant role as targeted therapies in nanomedicine, most especially for life-threatening diseases such as cancer^2^.

*Gunnera. perpensa* is the most popular *Gunnera* species in Sub-Saharan Africa. The earliest species of the genus, *Gunnera perpensa,* was described by Linnaeus in 1767 and is found throughout Africa, including the Democratic Republic of the Congo (DRC), Burundi, Ethiopia, Kenya, Tanzania, Botswana, Namibia, Zimbabwe, Mozambique, Lesotho, South Africa, and Swaziland^3,4^. The great therapeutic value of *Gunnera perpensa* in numerous traditional medical systems in southern Africa has led to the development of some formulae or prescriptions. Decoctions or infusions prepared with *Gunnera perpensa* as the main ingredient are sold in supermarkets, pharmacies, and unofficial markets such as muthi shops^4^. Moreover, many of these formulations or prescriptions are currently employed in clinical settings*.* Different ethnic groups in southern Africa have long utilized *Gunnera perpensa* as a concoction to induce labor, assure a simple delivery, and assist the placenta's ejection and cleaning of the womb following birth in both humans and animals^4–7^. Furthermore, in the Southern African countries, Lesotho and South Africa, warm aqueous infusions and decoctions of *Gunnera perpensa* are administered orally for three to four weeks for the treatment of cancer^4,8^.

The successful clinical application of the three-generation platinum anticancer drugs, cisplatin, carboplatin, and oxaliplatin, has promoted research interest in metallodrugs; however, the problems of drug resistance and adverse effects have hindered their further application and effects^9^. Thus, scientists are searching for new anticancer metallodrugs with lower toxicity and higher efficacy. The environmentally safe and sustainable method of producing ruthenium oxide nanoparticles (RuONPs) using green synthesis holds great potential for a range of applications. RuONPs produced by green synthesis have special qualities such as regulated shape, biocompatibility, synergistic effects, and the ability to generate ROS^9^, which makes them appealing for targeted cancer therapy. Ruthenium complexes have emerged as the most promising alternatives to platinum-based anticancer agents because of their unique multifunctional biochemical properties^9^. Thus, in this study, *G. perpensa* was utilized for the green synthesis of ruthenium oxide nanoparticles, and its cytotoxicity against MCF7 (breast cancer cell line) and Vero (a non-cancerous cell line) was investigated. The phytochemical constituents reduce the ruthenium chloride salt. The main focus of this article was to report the characterization of green synthesized ruthenium oxide nanoparticles (RuONPs) and their potential cytotoxicity against the MCF7 cell line.

## Methodology

### Plant collection and extraction

Plants were collected from their natural habitat in Mohale’s Hoek, Lesotho, permit to collect the plant materials was issued by the Department of Tourism Environment and Culture. To be conservative, the leaves were used and not the roots, moreover, this plant is not part of the endangered species in Lesotho. A Botanist at the Botany Department, University of the Free State (UFS), authenticated the plant with the voucher specimen number GP002CUT. The leaves of *Gunnera perpensa* were washed thoroughly with double distilled water and dried for a week at room temperature. The dried and finely cut leaves (20 g) were boiled in a 250 ml Erlenmeyer flask with 100 ml of double distilled water for 30 min. Then the extract was filtered through ordinary Whatman No. 1 filter paper. The filtrate with a pH = 3.5 was collected and was kept in a refrigerator at 4 °C for further experiments.

### Synthesis of the nanoparticles

Synthesis of nanoparticles was conducted using an established method by Lefojane et al.^10^ with slight modifications. Five grams (5 g) of ruthenium chloride [RuCl_3_xH_2_O] (Sigma-Aldrich) was mixed with 30 ml of *Gunnera perpensa* leaves extract for the synthesis of ruthenium oxide nanoparticles. An immediate colour change to dark grey was observed, thereby indicating the reduction of Ru^3+^ to Ru^0^ as in ruthenium nanoparticles. Reduction of Ru ions takes place with the action of phytochemicals present in the leaf extracts. Complexation of reduced Ru^0^ and Ru^+/2+^ ions with phytochemicals^11^ (steroids, glycosides, flavonoids, tannins, etc.) in the aqueous extract of *Gunnera perpensa*, and H_2_O molecules yields complexes that are precursors to the formation of ruthenium oxides. The aqueous extract containing chelated ruthenium was left to stir at 60 °C on a hot plate for 24 h. The mixture was then dried for 2 days in an oven at 80 °C giving rise to a blue-tainted black powder.

### Characterization of ruthenium oxide nanoparticles

The characterization of synthesized RuONPs was conducted using an established method by Lefojane et al.^12^. Structural characterization for size and particle shape determination was done using Scanning Electron Microscopy (SEM). The X-ray diffraction was used to confirm the crystallinity, phase structure, and purity of ruthenium oxide nanoparticles. For chemical bond speciation of biomolecules within the plant extracts and the ruthenium oxide nanoparticles formed, Fourier Transform Infrared spectroscopy was used. Lastly, the absorbance of the powder containing ruthenium oxide nanoparticles was measured using ultraviolet–visible spectroscopy.

### Cell culture: screening of the nanoparticles for cytotoxicity

The culture environment was kept at 37 °C in a humidified, concentrated 5% carbon dioxide atmosphere. DMEM media, supplemented with 10% serum (FBS) was used to grow and incubate MCF7 and Vero cells (these ATCC cells were purchased from Highveld Biological, South Africa). When the cells reached approximately 90% confluency trypsinization was performed. Warm (37 °C) Trypsin–EDTA solution 2 ml aliquots were added for 2 min to detach the cells. Equal amounts of complete medium were added to neutralize trypsin EDTA. The cell Viability was then determined using a trypan blue staining solution, and cell concentration was counted by an automatic cell counter (Invitrogen). Cells (MCF7 and Vero) were grown in DMEM media, supplemented with 10% serum (FBS), and incubated in the culture environment. Trypsinization was performed when the cells reached approximately 90% confluency. Aliquots of 2 mL of warm (37 °C) trypsin–EDTA solution were added for 2 min to detach the cells. Ten trypsin–EDTA was neutralized by adding equal amounts of complete medium. The cell viability was determined by using a trypan blue staining solution, and cell concentration was counted by an automatic cell counter (Invitrogen). The 96 well plates were used for seeding the cell suspension of 1 × 10^5^ cells/mL in aliquots of 100 µl, then the growth medium was added to each well, followed by incubation at 37 °C in a humidified 5% CO_2_ atmosphere for 24 h. After the 24-h incubation period, the medium was aspirated, and the cells were treated with 100 µl of a range of dilutions (100–0.001 µg/mL) of nanoparticles and other control samples, in triplicates. A final volume of 200 µl was reached by adding aliquots of 100 µl of media. The incubation of plates took 48 h^2^. Cell growth and metabolic activity were measured using the MTT assay as described by Kumar et al.^13^. Experiments were repeated in triplicates in parallel with the standard drug. Excel was used to analyse percentage growth inhibition.

## Results and discussion

### Crystallinity: X-ray diffraction

**Figure 1:**
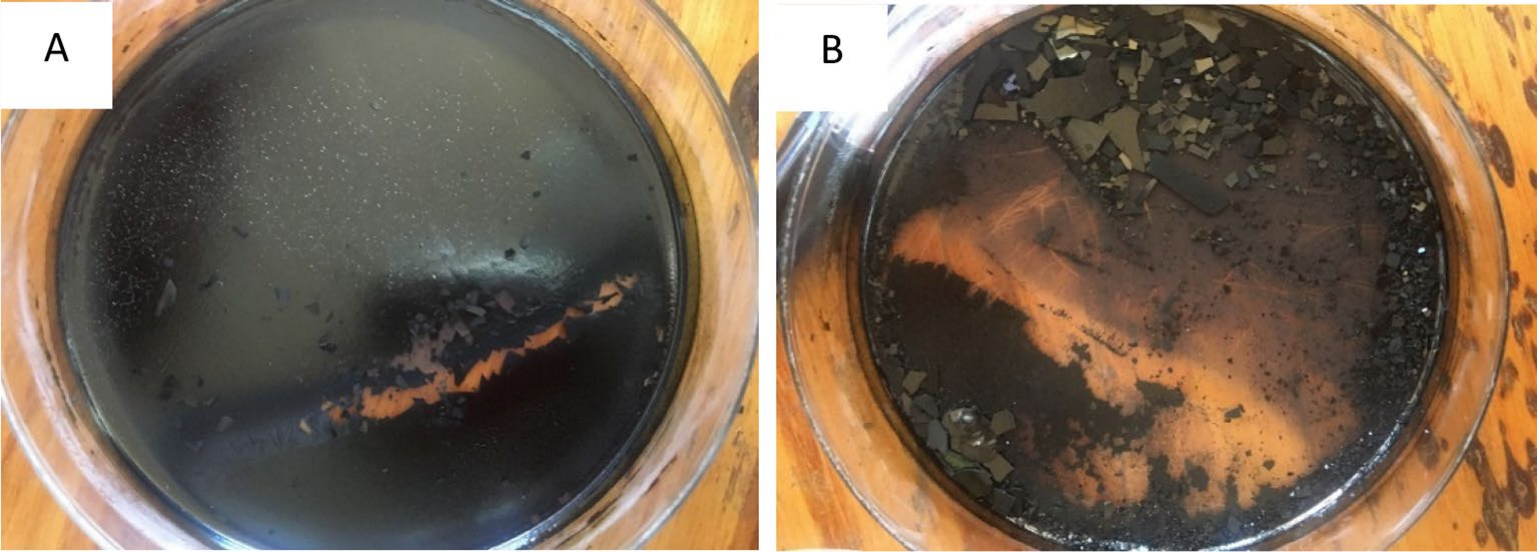
(**A**) Shows the ruthenium oxide nanoparticles in the oven while drying, and (**B**) after drying.

**Figure 2:**
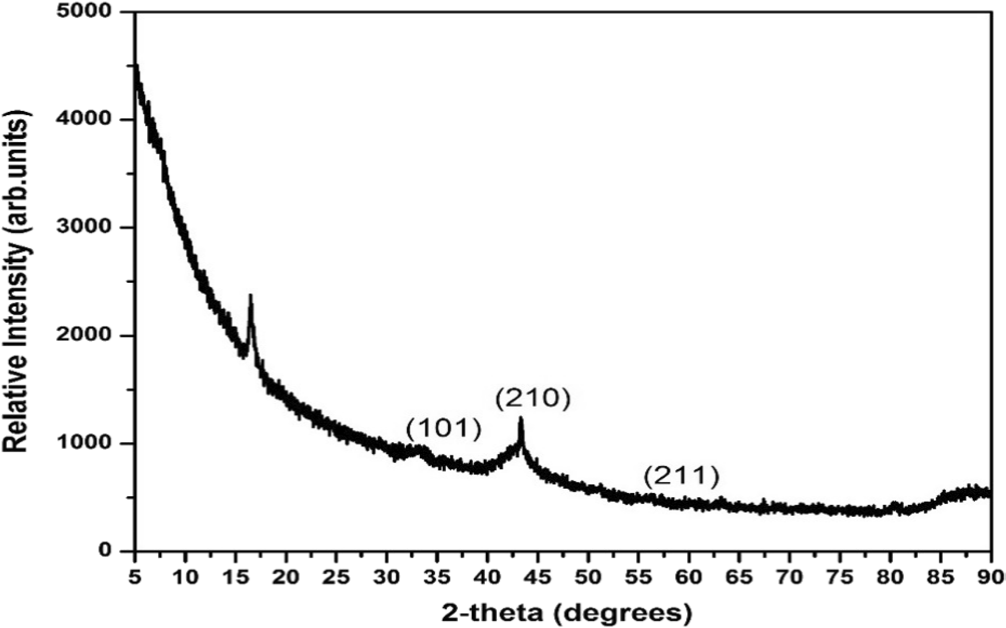
X-ray diffraction of the semi-amorphous RuONPs obtained after drying the RuO-*Gunnera perpensa* extract at 60 °C for 24 h and 80 °C for 2 days.

After the drying of ruthenium oxide nanoparticles, the blue-black crystals were also observed and shown in Fig. 1B. Figure 1A,B below shows the picture of ruthenium oxide nanoparticles in the process of drying. In this study, a Malvern PANalytical X’PERT PRO X-ray diffractometer with Cu Kα radiation of wavelength, λ = 0.15406 nm was used to determine the crystallinity of the powder prepared, its purity, and the structural phases in which it crystallized. XRD analysis (Fig. 2) showed the existence of crystalline RuO_2_ in a semi-amorphous state^14^, suggesting the formation of nanoparticles of RuO_2_. Bragg reflections at 33.18, 43.32, and 63.3° 2-theta with Miller indices (101), (210), (211) can be attributed to tetragonal RuO_2_ (JCPDS# 88-0322). The Bragg reflection observed at 16.49° 2-theta, we attribute to the presence of residual RuCl_3_/the formation of Ru-O-Cl^15^. The increase in background radiation observed at low 2-theta angles in the XRD pattern can be attributed to the scattering of radiation in the air, the geometry used for measurements, and some possible contributions from the sample which is in a semi-amorphous state.

The solubility of amorphous materials is much higher than that of crystalline^16^. An increase in solubility is significant as it can improve biopharmaceutical performance^14,16^. To estimate the average crystallite size of the RuONPs obtained after drying the Scherrer equation (Eq. 1) was used:

1 $$ {\text{D}} = K\lambda /\beta \cdot {\text{cos }}\left( \Theta \right), $$

wherein D is the particle size in nm, λ is the X-ray wavelength (Å), *K* is the shape factor, and Θ is half the Bragg angle, *β* is the Full Width at Half Maximum of the selected peak (in radians). Assuming a spherical shape (which is a generous approximation of the shape of RuONPs obtained in this work), with K = 0.94, and using the (210) peak the crystallite size was calculated to be 7.31 nm. The crystallite size of RuCl_3_/RuOCl impurity was calculated to be 0.24 nm.

### Morphology: scanning electron microscopy

**Figure 3:**
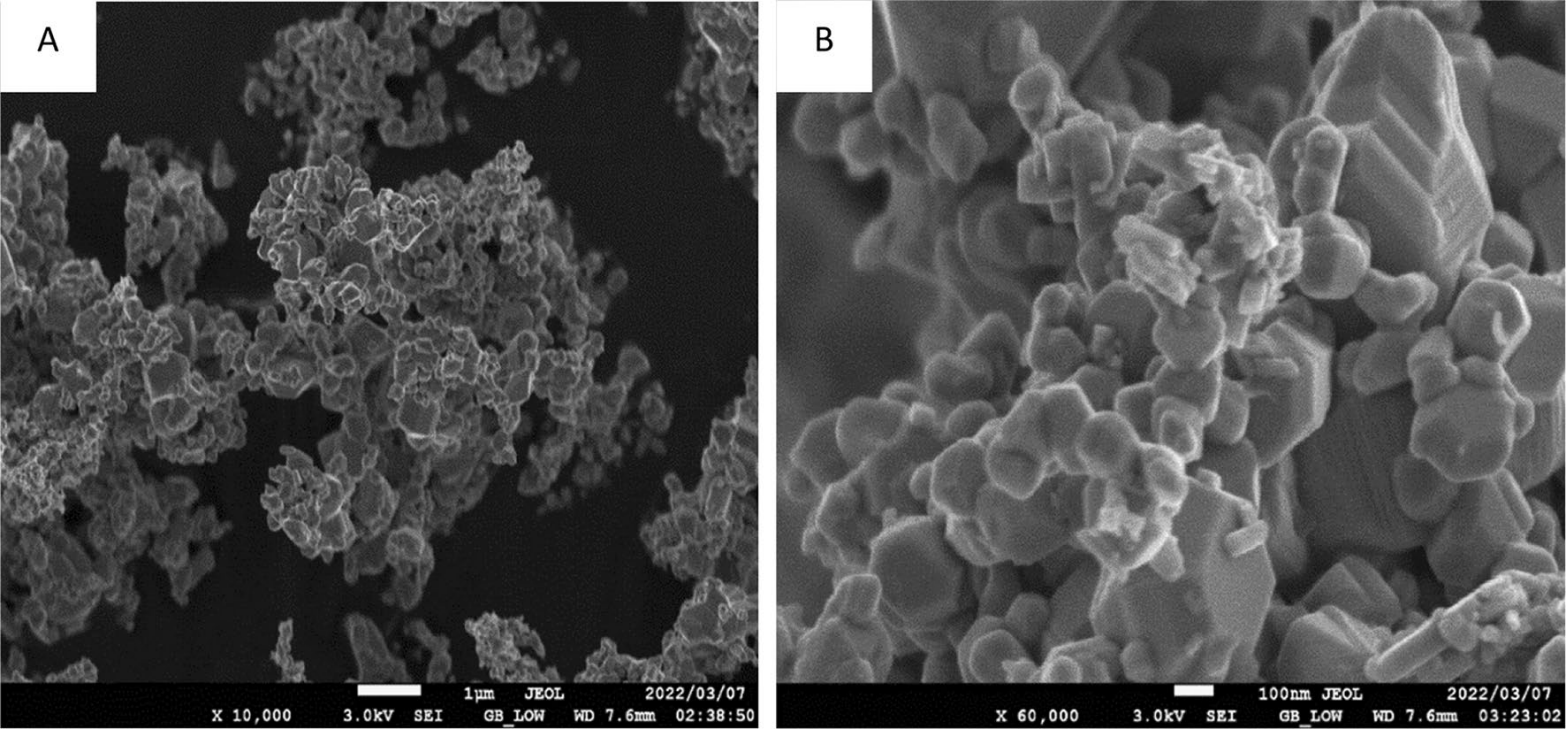
SEM micrographs show the morphology of ruthenium oxide nanoparticles at different magnifications (**A**) ×10,000; (**B**) ×60,000.

The structural morphology of synthesized ruthenium oxide nanoparticles was determined by Scanning Electron Microscopy. At a lower magnification of 10,000× (Fig. 3A), RuO nanoparticles were observed to be agglomerated. Higher magnifications at 60,000× in Fig. 3B showed that the ruthenium oxide nanoparticles were made of hexagonal-like platelets and giant octahedral cuboid-like structures at magnification. Agglomeration of the RuONPs is likely to result in a reduction in surface area and active sites available for interaction with cancer cells. Agglomeration coupled with the complexation of the Ru ions by biomolecules in the *Gunnera perpensa* plant extract may result in reduced activity of the RuONPs produced by this method of green synthesis. These two factors may account for the lower anti-cancer activity observed with the RuONPs when compared to Doxorubicin and Ru metal.

### Absorbance: ultraviolet–visible spectroscopy (UV–VIS)

**Figure 4:**
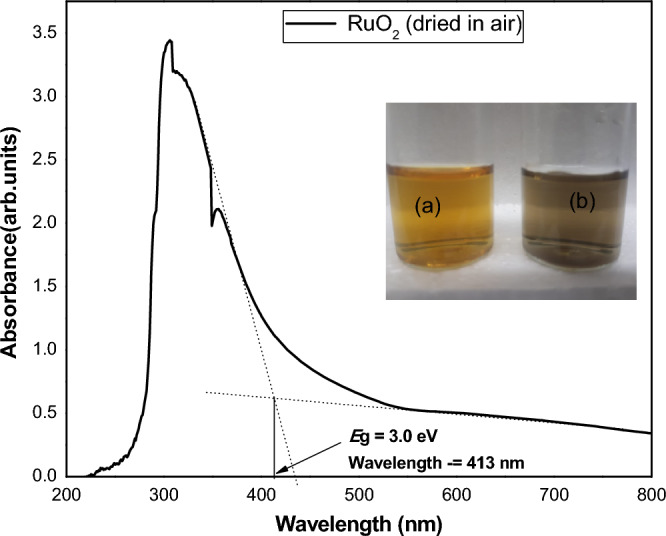
Ruthenium oxide NPs UV–Vis spectrum showing the absorption edge at 413 nm. Inset (a) *Gunnera perpensa* extract in deionized water and (b) RuONPs (consisting of RuO_2_) dissolved in deionized water.

A Cary 5000 UV–VIS-NIR spectrophotometer was used to measure the absorbance of the synthesized RuONPs by dissolving the RuONPs in deionized water in a cuvette with a path length of 1 cm. Typically bulk RuO_2_ is metallic^17^. It should therefore absorb well into the Visible/IR region of the electromagnetic spectrum. Nanoparticles of RuO_2_ are reported in the literature to exhibit semiconductor properties and absorb between 1.0 and 2.2 eV (2.7 eV for RuO_2_ quantum dots)^17,18^. The absorbance peak observed (Fig. 4) shows that RuONPs prepared in this work absorb at the boundary of the UV/Vis region. Reduction of particle size from bulk to nanoscale (crystallite size = 7.31 nm) which results in the widening of the bandgap due to a quantum confinement effect would be observed as absorption in this UV/Vis region^19^. However the presence of residual RuCl_3_ in the RuONPs obtained after drying the RuO/*Gunnera perpensa* extract might contribute to the absorption. The predominance of the RuO_2_ in the sample is however made evident by the absorption at the boundary of the UV–Vis and the black colour.

The profile of the UV–Vis absorption spectrum for RuONPs is akin to that reported by Anjum and co-workers^19^, in which they ascribe absorption at the UV–Vis boundary to RuO_2_ nanoparticles which they synthesized by annealing, at 600 °C, powders obtained from the plant-mediated reduction of RuCl_3_.xH_2_O using *Moringa olifeira* and *Catharantus roseus*. Based on the UV–Vis absorption spectrum in Fig. 4 and the assumption that the contribution of the RuCl_3_ impurity in the RuONPs is minimal the optical band gap (*E*_g_) of what is predominantly RuO_2_ in a semi-amorphous state is estimated to be 3.00 eV using Eq. (2) where

2 $$ E_{{\text{g}}} = hc/\lambda , $$

and in which *h* = Planck’s constant, *c* = speed of light, λ = wavelength at the absorption edge. In terms of electron volts E (eV) = 1240 (eV.nm)/λ (nm) = 1240 eV.nm/413 nm = 3.0 eV. The value of 3.0 eV is not far off from the value of 2.7 eV reported by Parveen and coworkers for RuO_2_ quantum dots^18^. The contribution of residual RuCl_3_ to the calculated band gap of the RuONPs prepared in this work should however not be overlooked. The closeness of the value to that of RuO_2_ quantum dots^19^ (particle size = 3 nm) points however to the predominance of RuO_2_ in the RuONPs powder.

### Chemical bond speciation using Fourier transform infrared spectroscopy (FTIR)

**Figure 5:**
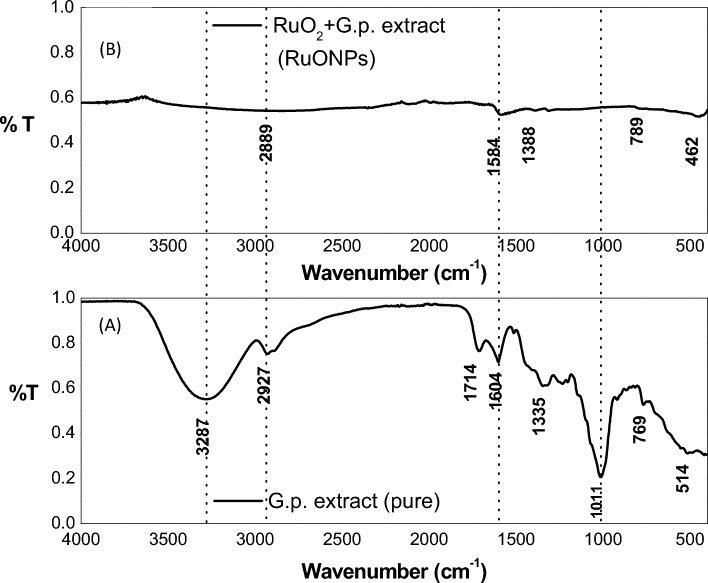
(**A**) FTIR spectrum of pure *Gunnera perpensa* extract after filtering and drying in air for 14 h (**B**) FTI-R spectrum of RuONPs after drying in air 60 and 80 °C for a period of 24 h and 2 days respectively.

FT-IR spectroscopy was used to determine the chemical bonds present in the plant extract as well as those present in the prepared RuONPs. The IR absorptions for RuO_2_ have been calculated to be in the region of 466 cm^−1^, 669 cm^−1^, 1019 cm^−1^, 1648 cm^−1^ and 3408 cm^−1^^20^. In comparison vibrations at 462, 1630 and 2889 cm^−1^ in Fig. 5B can therefore be attributed mainly to RuO_2_ whose unit cell is made up of 2 Ru atoms and 4 Oxygen atoms (Z = 2, space group *P*4_2_/*mnm*), with a geometry of 4 long bonds Ru–O and 2 short Ru=O bonds^21^. Vibrations at 1338, 1584 cm^−1^ (due to in-plane CH_2_-bending), and 2889 cm^−1^ (CH_2_-bending and possible residual OH-stretching from waters of crystallization present in Ru hydrates) can be attributed to the interactions between RuO_2_ and residual organics still present in the RuONPs.

In Fig. 5A the 514, 769, and 1101 cm^−1^ peaks depict C–H bending vibrations in phytochemicals present in the plant extract^22^. The peak at 2927 cm^−1^ can be ascribed to CH_2_– stretching vibrationsl^11^, while the strong broad peak at 3287 cm^−1^ points predominantly to the presence of H_2_O in the dried extract but also OH– stretching bonds found in the flavonoids, and steroids present^23^. The duplet at 1604 and 1714 cm^−1^ could be attributed to the presence of carbonyl groups (C=O) as may be found in steroids and flavonoids which are reportedly present in *Gunnera perpensa*.

It can be observed from Fig. 5A and B that peaks observed in the *Gunnera perpensa* extract are largely absent in the RuONPs—notes the almost complete disappearance of the 3287 cm^−1^ (H_2_O) 2927 cm^−1^ (CH_2_– stretching vibrations), a duplet at 1714 cm^−1^ (C=O), 1335 cm^−1^ (in-plane bending of CH_2_-groups), and 1011 cm^−1^. This suggests that: (1) much of the aqueous extract has effectively been separated from the RuO_2_ and any other Ru-complex formed both by decanting and drying off; (2) Ru metal and Ru ions in the Gunnera *perpensa* extract have effectively been complexed (chemically bound) to the phytochemicals present in the plant extracts; (3) the sample is quite dry (the strong broad peak at 3287 cm^−1^ observed in Fig. 5A due to water is almost completely absent in Fig. 5B).

### Cytotoxicity activity of ruthenium oxide nanoparticles

Synthesizing nanoparticles from plant-derived materials for anticancer activity presents a promising paradigm shift in cancer treatment. The synergistic combination of plant compounds and nanotechnology has the potential to significantly enhance therapeutic outcomes, minimize side effects, and contribute to the development of more effective and patient-centered cancer therapies. The phenol, and flavonoids in the plant extract are converted to the corresponding aldehydes, carboxylic acids, ketones, and flavones during the plant-mediated production of metal nanoparticles, while the metal ions are reduced to form metal nanoparticles. Through the targeted delivery of medicines to cancer cells, Nano formulations can increase the effectiveness of Ru complexes in the treatment of cancer while minimizing side effects and systemic toxicity. Cell growth inhibitory activity of ruthenium oxide nanoparticles was evaluated on MCF7 (hormone receptor-positive breast cancer cell line) and doxorubicin was used as the standard drug for cytotoxicity and anti-cancer activity, respectively. Vero cells (kidney non-cancerous cell line) were used for selectivity.

**Figure 6:**
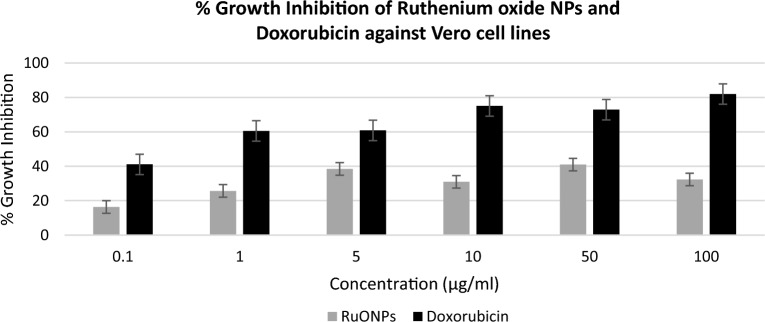
Depicts the comparison of %Growth Inhibition of RuONPs vs the standard drug (Doxorubicin) against the Vero cell line. Tests were repeated in triplicates and data was represented as mean ± standard deviation.

**Figure 7:**
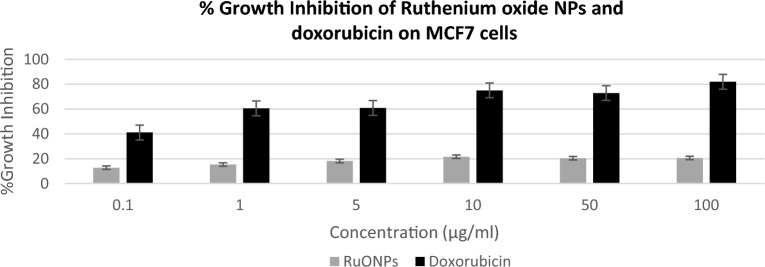
Depicts the comparison of RuONPs vs Standard drug (Doxorubicin) against MCF7 cell line. Tests were repeated in triplicates and data was represented as mean ± standard deviation.

Figure 6 shows that ruthenium oxide nanoparticles had the highest inhibitory activity of 22% at 10 µg/ml and the lowest inhibitory activity of 13% at 0.1 µg/ml. Our results showed that Vero cells and MCF7 cells viability were affected by synthesized ruthenium oxide nanoparticles, however, the inhibition was not selective. The percentage inhibition of doxorubicin on MCF7 ranges from 60 to 80% from a concentration range of 1 µg/ml to 100 µg/ml (as shown in Fig. 7).

The results from this study show that the ruthenium oxide nanoparticle's inhibitory activity against MCF7 cell lines was high when compared to the standard drug. The cytotoxicity of RuONPs was dose-dependent and the highest cytotoxicity was on MCF7 cells from the lowest concentration of 0.1 µg/ml. In addition, Fig. 7 shows the highest percentage growth inhibition of 59% as only 41% of cells were still viable after treatment with the standard drug at 0.1 µg/ml. while the lowest inhibition of more than 50% of the MCF7 cells was at 0.1 µg/ml for RuONPs and it was significant as compared to the control (p < 0.001). The IC50 value of RuONPs on both Vero and MCF7 cells was at 0.1 µg/ml, thus showing that RuONPs are highly cytotoxic to both Vero (non-cancerous) and MCF7 (breast cancer) cell lines.

A promising and novel anticancer drug should be selective in inhibiting cancer cells with minimal to no effect on the normal proliferation of non-cancerous cells. In this study, results show that RuONPs synthesized from *Gunnera perpensa* have a higher inhibitory activity on Vero cells, and thus could be considered cytotoxic to non-cancerous cells. Thus, suggesting that ruthenium oxide nanoparticles are not a good anti-cancer drug lead as they do not have selective cell inhibitory activity. According to Anjum et al.^24^, ruthenium as a compound has an anti-cancer activity, even though other studies showed the broad diversity of these compounds, in terms of activity, toxicity, and mechanisms of action^25^. Research findings by Liang et al.^26^, suggested that a compound (polypyridine ruthenium (II)) containing ruthenium has a high anticancer efficacy. Moreover, ruthenium (III) complexes have been reported to have a selective inhibitory effect on breast cancer cell lines and their mechanism of action is through the induction of apoptosis^27^. Contrary to this, ruthenium oxide nanoparticles synthesized from *Gunnera perpensa* did not show any selective activity. The isolated compounds from *Gunnera perpensa* have been reported to have antitumor activity on human breast cells in vitro^28^ and the crude extracts of this plant showed no cytotoxic activity on hepatic cells^29^. It should be noted that due to the fundamental nature of the synthesis process, existing approaches for making nanoparticles occasionally produce particles that lack selectivity^30^. The synthesis process may lead to the formation of non-selective nanoparticles with a wide size distribution and a variety of surface characteristics^30^. Thus, their efficacy in specific applications, such as medication delivery or cancer therapy, may be constrained by this non-selectivity. It should also be noted that there synthesized nanoparticles formed agglomeration as shown in Fig. 3. It is known that agglomeration/aggregation reduces the efficiency of nanoparticles and ultimately leads to subpar sample qualities^31^, thus, this could be another reason for the activity observed. From this study, it can be deduced that the combination of *Gunnera perpensa* and ruthenium oxide nanoparticles does not significantly enhance the anticancer activity of this plant as the ruthenium loses its selectivity.

## Conclusion

The different characterization techniques employed in this study show that the ruthenium oxide nanoparticles have been successfully synthesized. Characterization of nanoparticles is essential before toxicity studies can be done as the physical properties of nanoparticles influence toxicity to cancer cells. RuONPs have a high percentage inhibition on both Vero and MCF7, and they are not good anti-cancer drug leads. However further studies on RuONPs toxicity can be done with the focus on employing a chemical reduction method for synthesis or used as a carrier for chemo drugs because of their low inhibition on Vero cells.

## Data Availability

All data generated or analyzed during this study are included in this published article [and the supplementary information file].
